# Characterization of the HIV-1 integrase chromatin- and LEDGF/p75-binding abilities by mutagenic analysis within the catalytic core domain of integrase

**DOI:** 10.1186/1743-422X-7-68

**Published:** 2010-03-23

**Authors:** Yingfeng Zheng, Zhujun Ao, Kallesh Danappa Jayappa, Xiaojian Yao

**Affiliations:** 1 Laboratory of Molecular Human Retrovirology, Department of Medical Microbiology, Faculty of Medicine, University of Manitoba, 508-745 William Avenue, Winnipeg R3E 0J9, Canada

## Abstract

**Background:**

During the early stage of HIV-1 replication, integrase (IN) plays important roles at several steps, including reverse transcription, viral DNA nuclear import, targeting viral DNA to host chromatin and integration. Previous studies have demonstrated that HIV-1 IN interacts with a cellular Lens epithelium-derived growth factor (LEDGF/p75) and that this viral/cellular interaction plays an important role for tethering HIV-1 preintegration complexes (PICs) to transcriptionally active units of host chromatin. Meanwhile, other studies have revealed that the efficient knockdown and/or knockout of LEDGF/p75 could not abolish HIV infection, suggesting a LEDGF/p75-independent action of IN for viral DNA chromatin targeting and integration, even though the underlying mechanism(s) is not fully understood.

**Results:**

In this study, we performed site-directed mutagenic analysis at the C-terminal region of the IN catalytic core domain responsible for IN/chromatin binding and IN/LEDGF/p75 interaction. The results showed that the IN mutations H171A, L172A and EH170,1AA, located in the loop region _170_EHLK_173 _between the α4 and α5 helices of IN, severely impaired the interaction with LEDGF/p75 but were still able to bind chromatin. In addition, our combined knockdown approach for LEDGF/p75 also failed to dissociate IN from chromatin. This suggests that IN has a LEDGF/p75-independent determinant for host chromatin binding. Furthermore, a single-round HIV-1 replication assay showed that the viruses harboring IN mutants capable of LEDGF/p75-independent chromatin binding still sustained a low level of infection, while the chromatin-binding defective mutant was non-infectious.

**Conclusions:**

All of these data indicate that, even though the presence of LEDGF/p75 is important for a productive HIV-1 replication, IN has the ability to bind chromatin in a LEDGF/p75-independent manner and sustains a low level of HIV-1 infection. Hence, it is interesting to define the mechanism(s) underlying IN-mediated LEDGF/p75-independent chromatin targeting, and further studies in this regard will help for a better understanding of the molecular mechanism of chromatin targeting by IN during HIV-1 infection.

## Background

The human immunodeficiency virus type 1 (HIV-1) protein integrase (IN) catalyzes the insertion of proviral DNA into host chromosomes, a unique and obligatory step for all retroviral infection. The integration of proviral DNA is a two-step process involving 3' processing and 5' strand transfer, which has been well characterized by *in vitro *studies [1,2]. Integration occurs between a large nucleoprotein complex, referred to as the preintegration complex (PIC), and host chromatin. Nevertheless, how the PIC and chromatin interact within the nucleus remains largely unknown. Shortly after viral entry, the PIC formed in the host cellular cytoplasm is a functional nucleoprotein complex in which newly reverse transcribed viral DNAs are complexed with both viral proteins, including IN, matrix (MA), nucleocapsid, reverse transcriptase (RT), viral protein R (Vpr) and various cellular proteins (reviewed by Al-Mawsawi LQ et al.) [3]. These cellular proteins include Lens epithelium-derived growth factor (LEDGF), Integrase interactor 1 (INi1), high-mobility group protein 1 (HMGA1), barrier to auto-integration factor (BAF), Heat shock protein 60 (HSP60), *Polycomb *group embryonic ectoderm development (EED) protein, etc. (for a review see [4]). After nuclear import, PICs are targeted to the chromatin until successful integration into one of the host chromosomes.

As a functional component of PICs [5,6], the roles of LEDGF or p75 during lentiviral DNA integration have attracted increasing interest in recent years. LEDGF/p75, discovered as a general transcriptional co-activator [7], was isolated from a human lens epithelial cell (LEC) cDNA library and named LEDGF by Singh DP et al. [8]. LEDGF/p75 interacts with IN by its Integrase Binding Domain (IBD) (residues 341-429) [9,10]. The binding sites for LEDGF/p75 in IN are mainly located within the catalytic core domain (CCD) and around amino acids W131, W132 and I161-E170 [9,11-13]. The LEDGF/p75 plays multiple roles during HIV-1 infection through interaction with IN, such as protecting IN from proteasomal degradation [5], potentially affecting the nuclear transport of IN [5,14], stabilizing IN as a tetramer [15], enhancing IN enzymatic activities [16,17] and, most strikingly, serving as the IN-to-chromatin tethering factor driving PICs to transcriptionally active regions of host chromosomes [5,14].

A number of previous studies have employed *in vitro *biochemical approaches to study the interaction between IN and DNA substrates by using oligonucleotides that mimic the HIV LTR, and they have identified several residues in the IN that are responsible for its affinity for DNA [18-20]. All three domains of IN, including the N-terminal domain (NTD), CCD and C-terminal domain (CTD), have been shown to interact with DNA by *in vitro *studies [21-23]. However, how IN interacts with host chromatin under physiological conditions is considerably less well understood. Recently, by using a cell-based chromatin binding assay and co-immunoprecipitation (co-IP), we have identified three IN mutations (V165A, A179P, KR186,7AA) that impaired binding to host chromatin and LEDGF/p75 [24]. According to recent reports by Berthoux [25] and McKee et al. [15], the reduced affinity of IN KR186,7AA for LEDGF/p75 is due to disabled oligomerization of IN. As described previously, V165 is involved in the IN/LEDGF/p75 interaction interface [11,12,26], and A179 was identified as a new LEDGF/p75-binding site. The structure of the IN CCD and LEDGF IBD complex has been solved by a co-crystallization study [9]. Moreover, a recent study revealed that the interaction requires two asymmetric IN dimers and two LEDGF/p75 molecules, which was determined by mass spectrometry and cryo-electron microscopy [16]. However, both the architecture of the functional IN/LEDGF/DNA complex as well as the way in which these two proteins interact and work on both the viral DNA and host chromatin in the process of integration remain elusive. Further mutagenic analysis for IN/chromatin and IN/LEDGF interactions may not only help to elucidate the molecular mechanism of the IN/chromatin tethering and binding but also facilitate the identification of novel cellular factor(s) involved in this important viral replication step.

In the present study, we investigated the interactions of various IN mutants with host cell chromatin and LEDGF/p75 by cell-based chromatin binding and co-IP assays. In addition to previously described LEDGF/p75-binding defective IN mutants V165A, A179P, KR186,7AA [11,24,26], this study also identified several new IN mutants, including K159P, V176A and I203P, which reside in α4 to α6 helices of IN that lost the ability to bind to both chromatin and LEDGF/p75. Interestingly, we also found that several IN mutations, H171A, L172A and EH170,1AA, within the loop region _170_EHLK_173 _of IN, impaired the interaction with LEDGF/p75, but retained chromatin binding ability. This suggests that the IN is able to bind chromatin independently of LEDGF/p75. Consistently, our combined knockdown approach for LEDGF/p75 also failed to dissociate IN from chromatin. Moreover, we have also tested the effect of these IN mutants on HIV-1 infection, and our results revealed that the viruses harboring the IN mutants incapable of binding chromatin completely lost infectivity. However, viruses bearing IN mutants with chromatin-binding ability still sustained low levels of viral infection. All of these results clearly indicated that while the LEDGF/p75-binding ability of IN is important for productive HIV-1 replication, the IN has the ability to bind chromatin in a LEDGF/p75-independent manner and is sufficient to sustain a low level of HIV-1 infection.

## Materials and methods

### Cell lines and transfection

Human embryonic kidney 293T and the African green monkey kidney COS-7 cell lines were cultured in Dulbecco's Modified Eagles Medium (DMEM) supplemented with 10% fetal calf serum (FCS) and 1% penicillin-streptomycin. Human CD4+ C8166 T-lymphoid cells were maintained in RPMI-1640 medium supplemented with 10% FCS and 1% penicillin-streptomycin. For transfection of 293T cells and COS-7 cells, the standard calcium phosphate precipitation technique, was used as described previously [27].

### Plasmids and reagents

For chromatin binding, co-IP and immunofluorescence assay, various CMV-YFP-IN mutants including EH170,1AA, EK170,3AA, HL171,2AA and HK171,3AA were constructed by PCR-based site-directed mutagenesis. The nucleotide sequences of the sense mutagenic oligonucleotides are as follows: EH170,1AA, sense, 5'-AGATCAGGCTGCTGCTCTTAAGAC-3'; EK170,3AA, sense, 5'-GATCAGGCTGCACATCTTGCGACAGCAGT-3'; HL171,2AA, sense, 5'-AGGCTGAAGCTGCTAAGACAGC-3'; HK171,3AA, sense, 5'-AGGCTGAAGCTCTTGCGACAGCAGTAC-3'. The amplified HIV-1 IN fragment was cloned in-frame at the 3' end of the EYFP cDNA in a pEYFP-C1 vector (Clontech) at BglII and BamH1 sites. To construct pAcGFP-INwt/mut, each of the INwt/mut coding sequences was subcloned into pAcGFP1-C vector (Clontech) in-frame with the AcGFP coding sequence at BglII and BamH1 restriction sites. LEDGF/p75 was cloned into the pProLabel vector in-frame downstream of the ProLabel tag named pProLabel-LEDGF. SVCMVin-T7-LEDGF and the RT/IN/Env gene-deleted provirus (NL4.3Luc/ΔBg/ΔRI) were previously described [24,28]. To test the effect of different IN mutants on viral infection, cDNAs encoding for IN mutants, including EH170,1AA, EK170,3AA and HL171,2AA, were introduced into the SVCMV-Vpr-RT-IN expression plasmid by PCR-based method as described before [28].

Antibodies used for the immunofluorescence assay, immunoprecipitation or WB are as followed: the mouse monoclonal anti-β-Actin antibody (Abcam Inc.), rabbit anti-LEDGF/p75 (Bethyl Laboratories, Inc.) and anti-T7 monoclonal antibodies (Novagen), and a highly purified anti-GFP IgG fraction (through ion-exchange chromatography) purchased from Invitrogen Inc. (Cat. No. A6455) were used as primary antibodies. FITC-conjugated anti-rabbit antibody (Kirkegaard & Perry Laboratories (KPL)), anti-mouse (GE healthcare) and anti-rabbit HRP-conjugated antibodies (Amersham Biosciences) were used as the secondary antibodies.

### Chromatin binding assay

After transfection of YFP-INwt/mut into 293T cells for 48 h, the association of HIV-1 IN with cellular chromatin in mammalian cells was analyzed by a chromatin-binding assay [5,24]. To simplify the assay, only S1 (non-chromatin-bound) and S2 (chromatin-bound) fractions were analyzed by immunoprecipitation using an anti-GFP antibody and detected by WB with the same antibody. Protein bands in each fraction were further quantified with the software Quantity One (Bio-Rad), and the values are expressed as a percentage of chromatin-bound YFP-IN to total input, which consists of YFP-IN present in both S1 and S2.

### Immunofluorescence assay

COS-7 cells or 293T cells were grown on glass cover slips (12 mm^2^) in 24-well plates for 24 h and then transfected with different IN expression plasmids CMV-YFP-INwt/mut. After 48 h of transfection, cells on the cover slip were fixed and permeabilized for 30 min in methanol/acetone (1:1 ratio) at room temperature. Then, glass cover slips were incubated with primary rabbit anti-GFP antibody followed by secondary FITC-conjugated anti-rabbit antibody, and nuclei were stained with DAPI. Cells were visualized by a Carl Zeiss microscope (Axiovert 200) with a 63× oil immersion objective. To obtain the clearly defined intracellular localization of each YFP-INwt/mut, we adjusted the parameters of the imaging system for the best image of YFP-IN in glass slides.

### Co-immunoprecipitation assay and chemiluminescent co-immunoprecipitation (co-IP) assay

To detect the interaction between YFP-IN wt/mut and T7-LEDGF, the co-immunoprecipitation assay was carried out essentially as reported [24], except for modifications to the detection of the total input of YFP-IN and T7-LEDGF expression. Briefly, YFP, wild type YFP-IN or each YFP-IN mutant was co-transfected with T7-LEDGF into 293T cells for 48 h. The transfected cells were collected, and 90% of the cells were lysed in 0.25% NP-40 in 199 buffer supplemented with a cocktail of protease inhibitors and clarified by centrifugation at 13,000 rpm for 30 min at 4°C. The supernatant was subsequently subjected to IP with a rabbit anti-GFP antibody. The bound proteins were detected by WB using anti-T7 antibody. Meanwhile, 10% of transfected cells were lysed in 0.5% NP-40, and the lysates were used to detect the expression of YFP-INwt/mut and T7-LEDGF/p75 by WB using anti-GFP and anti-LEDGF antibodies, respectively.

The chemiluminescent co-IP assay was performed according to manufacturer's instructions. After AcGFP1-INwt/mut or AcGFP1-C and ProLabel-LEDGF fusion protein expression plasmids were co-transfected in 293T cells for 48 h, the cells were collected and lysed in 0.25% NP-40 lysis buffer and co-immunoprecipitated with Anti-GFP polyclonal antibody. For ProLabel detection of protein-protein interaction, the immunoprecipitates were resuspended in lysis/complementation buffer and transferred to a well in a 96-well assay plate (Costar, Corning, NY). To each well, the substrate mix was added, and ProLabel activity was measured using the POLARstar OPTIMA multidetection microplate reader (BMG Labtech).

### Transient knockdown of LEDGF/p75 in 293T cells

Duplex stealth RNA interference (RNAi) for LEDGF and scrambled RNAi were purchased from Invitrogen. 4 × 10^5 ^293T cells were seeded per well in a 6-well plate for 24 h and then cells were transfected with 20 nM siRNA oligonucleotides (Stealth RNAi; Invitrogen) directed against PSIP1/LEDGF/p75 mRNA using Lipofectamine 2000 (Invitrogen). Synthetic siRNA was designed with the following target and sequence: PSIP1HSS146003, targeting nucleotides 541 to 565 (5'UAAUGAAGGUUUAUGGGAGAUAGAU3'). In parallel, a scramble siRNA was used as negative control. The efficiency of LEDGF knockdown was monitored by WB at different time points (48 h, 72 h, 96 h, 120 h).

### The production and transduction of lentivirus vector containing LEDGF shRNA

To produce stable LEDGF/p75 gene knockdown 293T cell lines, the pLKO.1 lentiviral vector comprising siRNA hairpin targeting nucleotides of LEDGF/p75 mRNA was purchased from Open Biosystems. The hairpin structure contains a 21-bp stem, 5-nt loops, and 5' CCGG and 3' TTTTTG overhangs. The shRNA sequence RHS3979-97063117 targets the corresponding LEDGF/p75 mRNA nucleotides 860-880, and its stem-loop sequence was CCGGGCAGCTACAGAAGTCAAGATTCTCGAGAATCTTGACTTCTGTAGCTGCTTTTTG. The lentiviral particles harboring LEDGF/p75 shRNA were produced by co-transfecting the shRNA pLKO.1 vector, packaging DNA plasmid Δ8.2 and vesicular stomatitis virus G (VSVG) plasmid into 293T cells. After 48 h, supernatants containing lentiviral vectors were pelleted by ultracentrifugation (32,000 rpm at 4°C for 1 h) and stored in aliquots at -80°C.

To obtain stable LEDGF shRNA expressing cell lines, 293T cells were transduced with the shRNA LEDGF lentiviral vector for 48 h and then selected with 2 μg/mL puromycin for one week. Silencing of LEDGF/p75 was determined by WB analysis with an anti-LEDGF antibody. Detection of endogenous beta-actin was used for normalization of sample loading.

### Virus Production and Infection

A VSV-G pseudotyped single-cycle replicating virus was produced in 293T cells as described previously [24,28]. Briefly, 293T cells were co-transfected with an RT/IN/Env-deleted HIV-1 provirus NLlucΔBglΔRI, each CMV-Vpr-RT-IN (wt/mutant) expression plasmid and a VSV-G expression plasmid. After 48 h of transfection, viruses were collected and concentrated from the supernatants by ultracentrifugation at 35,000 RPM for 2 h. Virus titers were quantified using HIV-1 p24 Antigen Capture Assay Kit (purchased from the NCI-Frederick AIDS Vaccine Program). Equal amounts of viruses (adjusted by virion-associated p24 levels) were used to infect C8166 T cells overnight at 37°C. At 48 h post-infection, 1 × 10^6 ^cells from each sample were collected and lysed with 50 μL of luciferase lysis buffer (Fisher Scientific Inc). A 10 μL aliquot of cell lysate was subjected to the luciferase assay by using a POLARstar OPTIMA (BMG LABTECH, Germany), and the luciferase activity was valued as relative light units (RLU).

### Measurement of reverse transcription by quantitative PCR analysis

After production of the VSV-G pseudotyped single-cycle replicating viruses, equal amounts of virus (adjusted by virion-associated p24 levels) were treated with 340 IU/mL DNase (Roche Molecular Biochemicals) for 1 h at 37°C to remove residual plasmid DNA and then used to infect C8166 CD4+ T cells. For negative control (NC), prior to DNase treatment, wt virus was inactivated by incubating at 70°C for 0.5 h. The DNA was isolated from 1 × 10^6 ^C8166T cells at 12 h post-infection using QIAamp^® ^DNA blood kit (Qiagen sciences, Maryland, USA) following the manufacturer's instruction. The reverse transcription activity of HIV-1 in the infected cells was analyzed by quantifying the total HIV cDNA by using the qPCR technique. The qPCR was performed on Mx3000P detection system (Stratagene, CA) using LightCycler FastStart DNA Master SYBR Green I master mix (Roche diagnostics, Germany) along with forward (5'-tac tga cgc tct cgc acc-3') and reverse (5'-tct cga cgc agg act cg-3') primers targeted to the 5' end of the LTR and Gag region of the HIV-1 Bru genome [29]. The optimized thermal conditions used in the qPCR were as follows: initial hot start (95°C for 15 min) followed by 35 to 40 cycles of denaturation (94°C for 30 s), primer annealing (60°C for 30 s) and extension (72°C for 1 min). The total HIV-1 cDNA levels were expressed as copy numbers per cell, with DNA template normalized by the β-globin gene.

## Results

### Analysis of different HIV-1 IN mutants for their chromatin- and LEDGF/p75-binding

Our previous study showed that three IN CCD mutants V165A, A179P, KR186,7AA, which cannot bind LEDGF/p75, lack the ability to bind to host chromatin [24]. In the present study, we carried out a detailed mutagenic analysis to define binding site(s) for chromatin and LEDGF/p75 within the CCD of IN. Besides the previously reported IN mutants, V165A, A179P, KR186,7AA and a class I mutant D64/D116AA [24], several new YFP-IN mutants were generated by site-directed mutagenesis. The region E170-K173 was of interest because it overlaps with α-helices 4/5 connector residues 166-171 residing at the IN-LEDGF crystal interface [9]. Meanwhile, the mutagenic studies have highlighted the importance of E170A, H171A, LK172,3AA for LEDGF/p75 interaction [11,12,26]. The mutants K136, K159 were also included as they were reported to be involved in IN/nucleotide binding [30-32]. To address the role of α-helix 6 of IN in chromatin- and LEDGF interaction, mutants I200 and I203 were also included in the study. Table 1 lists 17 IN amino acid residues analyzed in the study, their conservations in different HIV-1 isolates, (the HIV sequences database was downloaded from the LANL website http://www.lanl.gov and aligned with MEGA4 program) and mutations introduced for each residue(s).

**Table 1:** Summary of IN mutant chromatin/LEDGF binding phenotypes

	Conservations *	Mutations	Chromatin binding	Interaction with LEDGF/p75
Wild type		Wild type	+++	+++
D64/D116	99.4/99.7	DD64, 116EA	+++	++
K136	31.3	K136A	+++	++
K159	99.5	K159P	-	+/-
V165	93.5	V165A	-	-
E170	99.6	H171A	+++	-
H171	98.5	L172A	+++	-
L172	99.4	EH170,1AA	++	-
K173	96.9	EK170,3AA	+++	+++
V176	99.4	HL171,2AA	-	-
A179	99.8	HK171,3AA	++	++
I182	98.0	V176A	-	-
F185	99.4	A179P	-	-
KR186,7	99.7/99.0	A179I	-	NA
I200	98.3	I182A	+	++
I203	96.8	F185A	+	++
		KR186,7AA	-	-
		I200A	**+**	-
		I203A	+++	++
		I203P	-	-

* Percent identify at that position among a collection of 1242 HIV-1 and SIVcpz strains http://www.hiv.lanl.gov.

**Figure 1:**
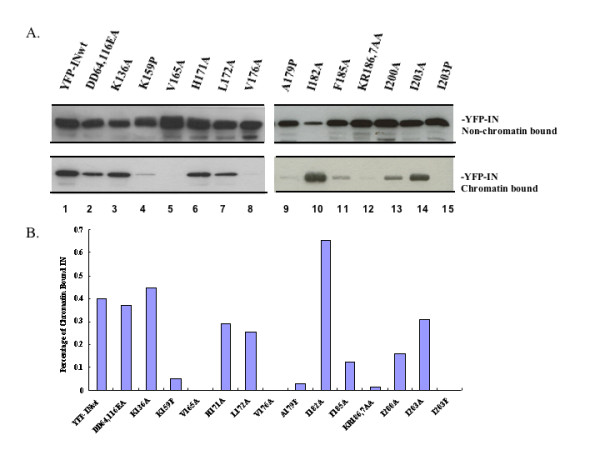
**Identification of chromatin binding sites within IN CCD**. A). 293T cells were transfected with different CMV-YFP-IN expressors (including the wild type IN and different mutants, as indicated). At 48 h post-transfection, cells were fractionated into chromatin-bound (lower panel) and non-chromatin-bound (upper panel) fractions as described in Materials and methods. YFP-IN in each fraction was analyzed by IP and WB with anti-GFP antibody. B). The intensity of both the chromatin-bound and non-chromatin-bound YFP-IN was densitometrically determined. The data are presented as the percentage of chromatin-bound YFP-IN to total input. Results are representative of two independent experiments.

These IN mutants were further subjected to the chromatin binding assay [24,33,34] to study their host chromatin binding ability. Briefly, each of YFP-INwt/mut was transfected into 293T cells, and, after 48 h, the presence of each YFP-INwt/mut in chromatin- and non-chromatin-bound fractions were analyzed by western blot with anti-GFP antibody, as described previously [24]. Our data showed that, in addition to the previously described IN mutants (V165A, A179P, KR186,7AA [24]) K159P, V176A, A179I, I203P were also severely impaired for host chromatin binding (Fig. 1A, data not shown for A179I). By contrast, mutants K136A, H171A, L172A, I182A and I203A were still able to associate with chromatin. The chromatin binding affinity of F185A and I200A was reduced by approximately 60% of wild type IN (Fig. 1A).

**Figure 2:**
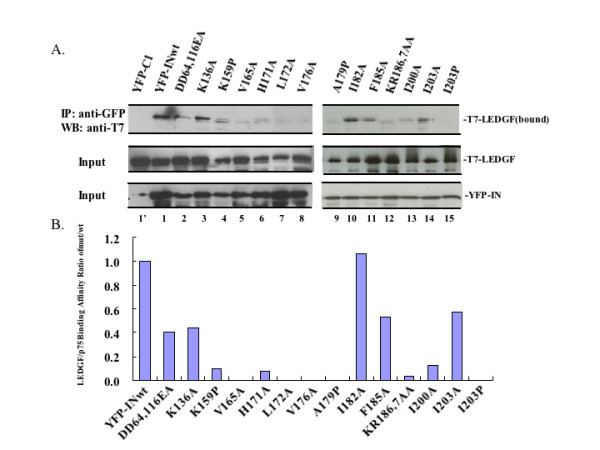
**Identification of LEDGF/p75-binding sites within IN CCD**. A). The CMV-YFP-INwt/mut or CMV-YFP plasmid was co-transfected with SVCMVin-T7-LEDGF expressor in 293T cells. After 48 h of transfection, 90% cells were lysed and subjected to co-IP assay. The IN bound T7-LEDGF/p75 was precipitated by using rabbit anti-GFP antibody and detected by WB using mouse anti-T7 antibody (upper panel). 10% cells were lysed with 0.5% NP-40, directly loaded on 10% SDS-PAGE gel and probed with anti-T7 or anti-GFP antibody to detect T7-LEDGF or YFP-IN expression (middle or lower panel). B). The intensity of protein bands was densitometrically determined. Results were expressed as the ratio of bound T7-LEDGF/p75 expression (mutants/wild-type) which was normalized by total input. Binding affinity to LEDGF/p75 of YFP-IN wild type was arbitrarily set as 100%. Results are representative of two independent experiments.

Because LEDGF/p75 has been shown to be involved in IN chromatin targeting, we also tested the LEDGF/p75-binding ability of different IN mutants by a cell-based co-IP assay. Equal amounts of T7-LEDGF and CMV-YFP-IN wt/mut plasmids were co-transfected into 293T cells. After 48 h of transfection, IN/LEDGF/p75 interaction was analyzed by co-IP of YFP-IN with anti-GFP antibody followed by Western blot (WB) with anti-T7 antibody. Results revealed a strong interaction between T7-LEDGF and YFP-IN wild type and mutants D64E/D116A, K136A, I182A, F185A, I203A. Meanwhile, the mutants K159P, H171A, and I200A showed reduced affinity for LEDGF/p75 (Fig. 2A, lanes 4, 6, and 13). Interestingly, several IN mutants including V165A, L172A, V176A, A179P, KR186,7AA, I203P lost their interaction with LEDGF (Fig. 2A. lanes 5, 7, 8, 9, 12, and 15). As expected, no T7-LEDGF/p75 was pulled down by YFP control (Fig. 2A, lane 1). To ensure that similar amounts of T7-LEDGF/p75 and YFP-IN were expressed in each sample, the presence of T7-LEDGF/p75 and YFP-IN in each sample was detected by WB with corresponding antibodies (Fig. 2A, middle and lower panel). The host chromatin and LEDGF/p75 cofactor interaction data of all the IN mutants analyzed in this study have been summarized in Table 1. Interestingly, we noted that IN mutants, H171A and L172A, displayed a drastically reduced interaction with LEDGF/p75 but still retained the interaction with chromatin.

### Chromatin- and LEDGF/p75-binding analysis of IN double mutants within Loop 170EHLK_173_

**Figure 3:**
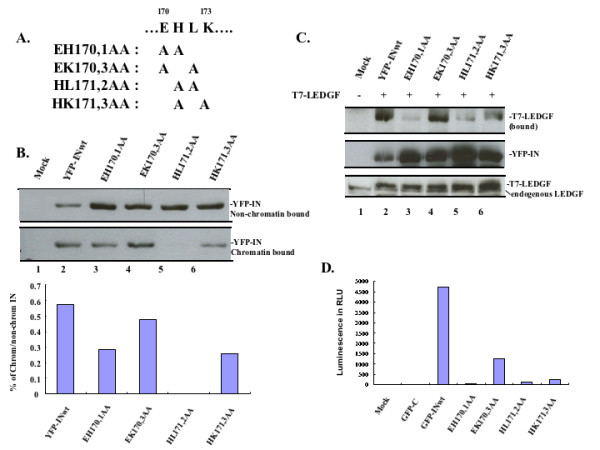
**Differential effects of IN mutations within _170_EHLK_173 _region on chromatin- and LEDGF-binding**. A). Diagram of amino acids sequence and introduced mutations in HIV-1 IN _170_EHLK_173 _domain. B). Chromatin binding profiles of IN double mutants within _170_EHLK_173_. 293T cells were mock-transfected or transfected with equal amount of CMV-YFP-IN wild type or double mutants EH170,1AA, EK 170,3AA, HL171,2AA and HK171,3AA. At 48 h post-transfection, cells were fractionated into chromatin-bound and non-chromatin-bound fractions as described in Materials and methods. YFP-IN in each fraction was analyzed by IP and WB with anti-GFP antibody. Chromatin binding affinity was quantified by laser densitometry and results are shown as the percentage of chromatin-bound to total input of YFP-IN (lower panel). C) LEDGF-binding affinity within IN _170_EHLK_173 _by co-IP assay. 293T cells were co-transfected with the SVCMVin-T7-LEDGF/p75 expressor and CMV-YFP-INwt/mut plasmid as indicated. After 48 h of transfection, 90% of cells were lysed and subjected to co-IP assay as described before. The upper panel showed the bound T7-LEDGF/p75 in each sample. 10% of cell lysates were used to detect the expression of YFP-INwt/mut and T7-LEDGF/p75 by WB using anti-GFP and anti-LEDGF antibodies respectively (middle panel and lower panel). D). LEDGF-binding affinity within IN _170_EHLK_173 _detected by chemiluminescent co-IP assay. AcGFP1-INwt/mut or AcGFP1-C and ProLabel-LEDGF fusion proteins were coexpressed in 293T cells. After 48 h of transfection, cells were lysed and immunoprecipitated with anti-GFP antibody and the chemiluminescent signals from ProLabel-LEDGF present in the complexes were measured by using ProLabel Detection Kit II and valued as relative luminescence units (RLU). Results are representative of two independent experiments.

Interestingly, two IN mutants, H171A and L172A, that showed differential binding abilities to chromatin and to LEDGF/p75 are located in the CCD loop region _170_EHLK_173 _of IN, a connector that links helices α4 and α5. Thus, we then focused our studies on this region, which may be important for LEDGF/p75-binding, but not for IN chromatin-association. Indeed, this region overlaps with the interface for LEDGF-binding in the crystal study [9], and some IN mutants within this region, such as E170A, H171A, and LK172,3AA, have been shown to be impaired in the ability to bind LEDGF/p75 [11,12,26]. To further elucidate the functional roles of loop _170_EHLK_173 _on its chromatin and LEDGF-binding, we characterized the binding affinities of this region by testing the double mutants YFP-IN EH170,1AA, HL171,2AA, EK170,3AA and HK171,3AA (Fig. 3A). The chromatin-association experiment showed that three of the double mutants EH170,1AA, EK170,3AA and HK171,3AA displayed strong binding affinity with cellular chromatin, whereas HL171,2AA completely lost its chromatin binding ability (Fig. 3B). Meanwhile, the LEDGF/p75-binding ability of each mutant was also tested by co-IP assay, and results showed that all the mutants except YFP-IN EK170,3AA lost their ability to interact with LEDGF/p75 (Fig. 3C). The differential LEDGF-binding abilities of these four IN double mutants were re-confirmed by chemiluminescent co-IP assay (Fig. 3D). Altogether, uncoupled chromatin- and LEDGF-binding affinities were observed for IN mutants H171A, L172A and EK170,1AA, with strong binding affinity to chromatin but dramatically impaired contact with LEDGF/p75.

### Nuclear localization of IN mutants in COS-7 cells

**Figure 4:**
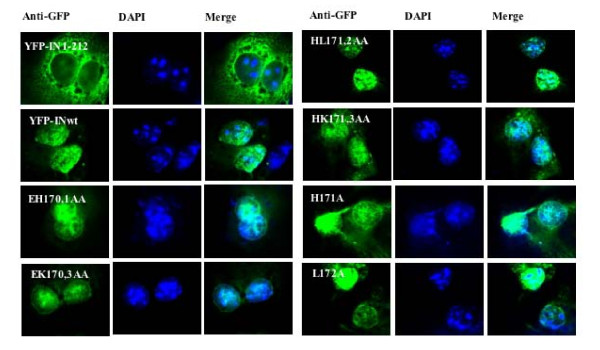
**Subcellular localization of IN _170_EHLK_173 _mutants in COS-7 cells**. COS-7 cells were transfected with different CMV-YFP-IN fusion protein expressors as indicated for 48 h. After fixation and permeabilization, cells were incubated with primary rabbit anti-GFP antibody followed by secondary FITC-conjugated anti-rabbit antibodies, and the nuclei were stained with DAPI. Cells were visualized by a Carl Zeiss microscopy (Axiovert 200) with a 63× oil immersion objective.

Since HIV-1 IN has been shown to be a karyophilic protein and is involved in nuclear import of PICs, we wondered whether introducing mutations in the _170_EHLK_173 _region of IN might interfere with IN nuclear translocation, which consequently affects their association with chromatin and/or LEDGF/p75 binding. To address this question, we transfected each IN mutant into COS-7 cells and analyzed their intracellular localization by immunofluorescence. Given the low expression of the YFP-IN fusion protein in COS-7 cells, the indirect immunofluorescence technique was used (as described in Materials and Methods). Results showed that, while the wild type IN was localized in the nucleus, the IN C-terminal deletion mutant YFP-IN1-212 was excluded from the nucleus, consistent with previous studies [24,28]. Also, all the IN _170_EHLK_173 _region mutants, including EH170,1AA, HL171,2AA, EK170,3AA and HK171,3AA, were able to accumulate predominantly in the nucleus (Fig. 4). All of these results indicate that 1) the _170_EHLK_173 _region is dispensable for IN nuclear localization; and 2) the LEDGF/p75- and/or the chromatin-binding defects of those IN mutants were not due to their impaired nuclear translocation.

### Knockdown of LEDGF/p75 had no effect on IN's chromatin binding

**Figure 5:**
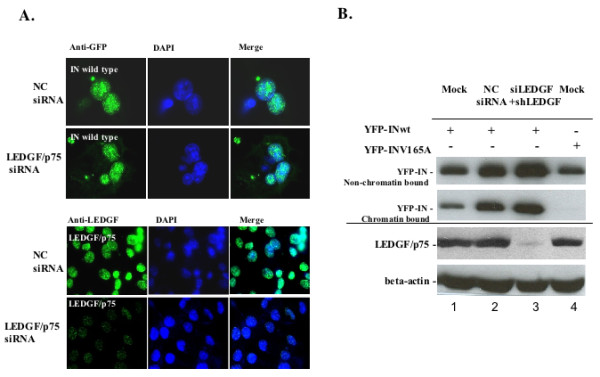
**LEDGF/p75 is not required for chromatin binding of IN**. A). Transient knockdown of LEDGF/p75 by siRNA had no effect on IN nuclear localization. 293T cells were transfected with either 20 nM negative control (NC) siRNA or 20 nM si-LEDGF PSIP1HSS146003 for 24 h before transfection with CMV-YFP-IN wild type. At 48 h post-transfection, cells were fixed, permeabilized and detected for YFP-IN and LEDGF/p75 expression by using anti-GFP or anti-LEDGF antibodies. The nuclei were stained with DAPI. B). Analysis of chromatin binding affinity of IN on LEDGF/p75 knockdown cells. The lentiviral shRNA-mediated LEDGF/p75 stable knockdown 293T cells were transfected with 20 nM si-LEDGF for 48 h and further transfected with YFP-IN wild type or mutant V165A and were analyzed for its chromatin binding affinity. In parallel, cells were either mock-transfected or transfected with negative control siRNA to study chromatin binding of YFP-IN wild type. The chromatin bound and non-chromatin-bound fractions of YFP-IN wild type or V165A were showed as indicated. The LEDGF/p75 expression level in each sample was verified by WB with anti-LEDGF antibody. Endogenous beta-actin was used for normalization of sample loading.

Uncoupled chromatin- and LEDGF-binding affinities observed in IN mutants within the _170_EHLK_173 _region suggest that LEDGF/p75 may not be essential for IN binding to chromatin. To gain more insight into the association between IN chromatin binding and IN/LEDGF interaction, we tested the effect of LEDGF/p75 knockdown (LEDGF/p75-KD) on IN chromatin binding affinity. To obtain high efficiency gene knockdown, both synthetic small interfering RNAs (siRNAs) and short hairpin RNAs (shRNAs) were combined in the study to knockdown LEDGF/p75 expression in 293T cells, as described in Materials and Methods. The results showed that such combined transient and stable LEDGF/p75-KD resulted in over 90% reduction of LEDGF/p75 expression (Fig. 5B, lower panel). Then, the nuclear localization of HIV-1 IN in LEDGF/p75-KD cells was analyzed by indirect fluorescence using anti-LEDGF antibody. As shown in figure 5A (lower panel), control cells transfected with scramble siRNA displayed abundant LEDGF/p75 protein expression. However, only a trace amount of LEDGF/p75 was detected in 293T cells transiently transfected with siRNA. Then, the cells were stained with anti-GFP antibody to visualize the localization of IN. Results showed that the wild type YFP-IN in transient LEDGF/p75-KD cells still accumulated in nuclei, suggesting that the LEDGF/p75-KD did not exert any significant effect on IN nuclear localization (Fig. 5A, upper panel).

Next, we checked whether LEDGF/p75 depletion has an effect on IN chromatin binding. To do so, the LEDGF/p75-KD 293T cells were transfected with YFP-INwt, and after 24 h of transfection, cells were treated with MG-132, a proteasome inhibitor, to prevent IN degradation. Cells were processed for IN chromatin binding analysis at 48 h post transfection, as described above. Of note, no significant difference in the IN chromatin association was observed between the LEDGF/p75 KD cell line and the mock-transfected cell control (Fig. 5B, upper panel). In parallel, the 293T cells transfected with the YFP-IN V165A mutant, which has been shown to be defective of chromatin binding, was used as a negative control [24]. Thus, our results demonstrated that the LEDGF/p75 KD could not abrogate IN chromatin binding.

### Effect of IN _170_EHLK_173 _mutants on HIV-1 infection

**Figure 6:**
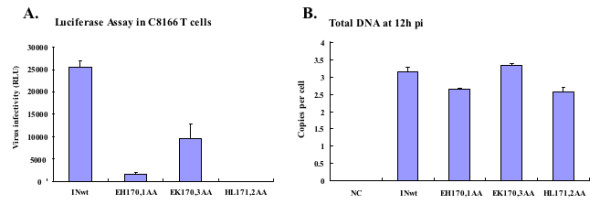
**A) The differential replication profiles of IN mutant viruses within the loop _170_EHLK_173 _on HIV-1 single-cycle replication**. 293T cells were co-transfected with an RT/IN/Env-deleted HIV-1 provirus NLlucΔBglΔRI, each CMV-Vpr-RT-IN (wt/mut) expressor and a VSV-G expressor to generate single round replication competent virus. To test the effect of different IN mutant viruses on HIV-1 infection, C8166 T cells were infected with equal amount of VSV-G pseudotyped IN mutant viruses (adjust by p24 level) for 48 h. 1 × 10^6 ^cells were collected and cell-associated luciferase activity was measured by luciferase assay at 48 h post-infection. B) Followed by 12 h infection with single cycle replicating viruses on dividing C8166 T cells, total DNA was extracted and amplified for total viral DNA and human β-globin gene using corresponding primers by Real time PCR. Total HIV-1 DNA levels were expressed as copy numbers per cell, with DNA template normalized by the amplification of the β-globin gene. NC: negative control or 70°C inactivated wt virus.

From the above results, we observed that LEDGF/p75 may not be mandatory for IN targeting to host chromatin. However, we still do not know whether LEDGF/p75 independent chromatin binding of IN could ensure HIV infection. To address this question, we introduced IN double mutants EH170,1AA, EK170,3AA, and HL171,2AA into an HIV-1 RT/IN trans-complemented single cycle replication system [24,35]. Briefly, each of these IN double mutants was first introduced into a CMV-Vpr-RT-IN expression plasmid. The VSV-G pseudotyped HIV-1 single cycle replicating viruses containing these individual IN double mutants and a luciferase gene, substituted for the Nef gene, were produced in 293T cells by co-transfecting each CMV-Vpr-RT-INwt/mut expression plasmid with RT/IN-deleted HIV provirus NLlucΔ Bgl/ΔRI, and a VSV-G expression plasmid. Then, the same amount of virus (normalized by p24 gag levels) was used to infect C8166 CD4+ T cells, and the level of infection was monitored by measuring the luciferase activity. The results showed that the mutant EK170,3AA, which can efficiently bind to both chromatin and LEDGF/p75, displayed about 30% replication capacity relative to the wild type virus (Fig. 6A). The chromatin-bound but LEDGF interaction defective IN mutant virus, EH170,1AA, induced a low level of infection, whereas the HL171,2AA mutant virus, which lost the ability to interact with both chromatin and LEDGF/p75, was non-infectious (Fig. 6A). Moreover, real time PCR analysis indicated that mutations introduced in the _170_EHLK_173 _did not significantly affect the reverse transcription step at 12 h post-infection (Fig. 6B). These data suggest that while IN/LEDGF/p75 interaction is important for a productive HIV-1 replication, the IN-mediated LEDGF/p75-independent chromatin binding is still able to sustain a low level viral infection.

## Discussion

While the interaction between IN and viral DNA was extensively investigated by *in vitro *studies [18,19,22,36-39], less was known for IN interaction with host chromatin under physiological conditions. Interestingly, a large number of recent studies have demonstrated that the cellular factor LEDGF/p75 plays an important role in tethering HIV-1 IN to the transcriptionally active units of host chromatin [40,41]. However, how IN alone, in the absence of LEDGF/p75, plays a role in chromatin binding needs to be fully understood. In this study, we performed site-directed mutagenic analysis at the C-terminal region of the IN CCD for IN/chromatin binding and IN/LEDGF/p75 interaction. Results showed that several IN mutants including K159P, V165A, V176A, A179P, KR186,7AA and I203P were unable to bind both LEDGF/p75 and host chromatin. The mutants H171A, L172A and EH170,1AA, located in a loop region _170_EHLK_173 _of IN, severely impaired their interaction with LEDGF/p75 but were still able to bind chromatin. Also, our data showed that LEDGF/p75 depletion in cells failed to dissociate IN from chromatin. Furthermore, the single-round HIV-1 replication assay results showed that the viruses harboring IN mutants capable of LEDGF/p75-independent chromatin binding still sustained a low level of infection. All of these data indicate that while LEDGF/p75 is important for productive HIV-1 replication, IN has the ability to bind chromatin in a LEDGF/p75-independent manner and sustain a low level HIV-1 infection.

The results showed that IN mutants K159P, A179P and I203P located at the α-helices 4, 5 and 6 specifically affected both chromatin- and LEDGF/p75-binding abilities. Since introducing proline often bends the amino-acid backbone and affects the secondary structure of the protein, it could be possible that introducing proline mutations disrupts α-helix formation and hampers both chromatin- and LEDGF/p75-binding abilities. Indeed, this could be the case for the IN mutant I203P because another mutant I203A was able to efficiently bind host DNA and LEDGF/p75 (Figs. 1 and 2 compare lane 14 to 15). However, given the fact that both A179P and A179I lost binding to host chromatin, the A179 residue may be directly involved in interacting with host chromatin (Fig. 1 and data not shown for A179I). Nevertheless, the chromatin-binding phenotype of K159P, A179P and I203P IN mutants suggest the involvement of α-helices 4, 5 and 6 of IN in host DNA recognition. Two other IN mutants that need to be addressed are KR186,7AA and F185A. We have previously shown that the IN mutant KR186,7AA was severely impaired in both chromatin- and LEDGF-binding affinities [24]. In this study, we identified another mutant F185A that displayed a significant reduction in the interaction with LEDGF and chromatin, but to a lesser extent than that of KR186,7AA. The K186 and R187 of IN, by crystallographic studies, are known to lie in the dimer-dimer interface of IN [42,43] and F185 has been implicated for tetramerization of IN [44]. So, mutations at F185, K186 and R187 might affect IN oligomerization and further impair its chromatin binding affinity. In addition, a recent study by Merad H et al. revealed that a helix-turn-helix (HTH) (residues 149-186) motif consists of two helices (helix 4 and helix 5) and that the loop in between is involved in recognition of viral DNA [19]. Interestingly, in our study, IN mutants K159A, V165A, V176A, A179P, KR186,7AA are located within this region and were identified as chromatin-binding defective mutants. Thus, the chromosomal attachment site within the IN CCD may also center on IN α-helix 4 to α-helix 5, and this HTH motif could be critical for the recognition of both viral and host DNA. However, how IN recognizes and binds both viral and host DNA sequence to form an active integration complex remains an open question and requires more detailed computational, experimental and structural investigations.

However, the functional roles of LEDGF/p75 and its potential correlation with chromatin binding of IN are of interest in our present study. It is well established that LEDGF/p75 serves as an IN-to-chromatin tethering factor, driving PICs to transcriptionally active regions of host chromosomes [5,14]. Our previous results showed that chromatin binding defective IN mutants (V165A, A179P, KR186,7AA) also fail to interact with LEDGF/p75, suggesting that LEDGF-binding of IN might be linked to the chromatin-binding affinity of IN [24]. Here, we attempted to select more IN mutants to map both chromatin- and LEDGF/p75-binding sites within the CCD of IN. Results showed that most of the IN mutants tested in this study lost both chromatin-binding and LEDGF/p75-interacting abilities, highlighting the importance of LEDGF/p75 as a tethering factor for IN chromatin targeting. Interestingly, two IN mutants, H171A and L172A within the CCD of IN, displayed a different phenotype; they could not efficiently interact with LEDGF/p75 yet still could bind chromatin (Fig. 1 and 2, see also Table 1). This raises the possibility that the HIV-1 IN may still be able to target chromatin in the absence of LEDGF/p75 association. Because H171 and L172 are located within or close to the loci of IN/LEDGF interface (α4/5 connector residues 166-171) [9], we next focused on detailed chromatin- and LEDGF-binding affinities within the IN region _170_EHLK_173. _For this purpose, four IN double mutants, EH170,1AA, EK170,3AA, HL171,2AA and HK171,3AA, were tested. Indeed, it was shown again that the IN mutant EH170,1AA showed relatively high affinity with host chromatin but was unable to bind LEDGF/p75 effectively, while the IN mutant HL171,2AA had defects on both the chromatin- and LEDGF-binding affinities (Fig. 3). These results suggest that the HIV-1 IN is able to bind chromatin independently of LEDGF/p75.

Because the IN mutants H171A, L172A and EH170,1AA bound to chromatin but not LEDGF/p75, we further reconfirmed the LEDGF/p75 independent chromatin binding of wild type IN using the LEDGF/p75-KD cells. Our results showed that the efficient knockdown of LEDGF/p75 had no significant effect on IN to chromatin-association, suggesting that the chromatin binding of IN might still take place in the absence of LEDGF/p75. Meanwhile, we ruled out the possible effect of LEDGF/p75 knockdown on nuclear translocation of HIV-1 with wild type IN by observing the intracellular localization of all the IN fusion proteins using immunostaining, which is indeed consistent with the previous observation [13]. Most likely, the IN is still able to target chromatin without preferential targeting sites in the absence of LEDGF/p75. Consistently, previous studies have highlighted that the role of LEDGF/p75 during HIV-1 integration is advantageous to HIV-1 integration but could be nonessential to the process of integration [5,45]. We speculate that, without the LEDGF/p75 tethering, IN might still be able to bind chromatin, but it might lack the preferential selection site. Also, it is possible that other unknown cellular factor(s) might contribute to the chromatin targeting of IN; such proteins should harbor both DNA-binding and IN-binding domains similar to that of LEDGF/p75. Further efforts are underway to seek new cellular partners involved in IN-to-chromatin targeting.

In an attempt to correlate IN chromatin-binding ability to its effect on virus infection, we introduced IN mutants EH170,1AA, EK170,3AA or HL171,2AA into a VSV-G pseudotyped HIV-1 single cycle replicating virus and investigated their effects on HIV-1 infection. As expected, viruses containing the IN HL171,2AA mutation, which lost both LEDGF/p75- and chromatin-binding abilities, are unable to replicate (Fig. 6). This result is consistent with previous reports in which the impaired integration of proviral DNA into host cell chromatin accounted for the replication defect of the L172 mutant virus [46,47]. Interestingly, another batch of viruses harboring the IN EH170,1AA mutation, which fail to associate with LEDGF/p75 but are still able to interact with chromatin, retain the infectivity towards the susceptible cell lines, although at a low efficiency. This suggests that the chromatin association of IN, rather than LEDGF/p75 binding, is essential for HIV-1 infection. These results are consistent with the previous study by Shun and his co-workers in which the LEDGF-null mouse embryo fibroblasts were able to support approximately 10% of HIV-1 integration compared to control cells [40]. These results again highlighted the importance of LEDGF/p75-binding property of IN during HIV-1 replication. It is possible that the LEDGF-independent chromatin binding of IN is still able to target viral PICs to host chromatin, but, without the escort of LEDGF/p75, such IN-mediated "nonspecific" chromatin binding is less efficient and/or could not efficiently target viral PICs to transcriptionally active sites in the chromatin and mediate a productive viral replication. Another interesting question is how IN is still able to interact with host chromatin under a very low level of LEDGF/p75. Whether it is through IN directly binding to host DNA or whether it requires other undefined cofactor(s) for this process remains unclear and requires more detailed study. Successful elucidation of the mechanism underlying how HIV-1 IN possesses a LEDGF/p75-independent chromatin binding and identification of other IN-interacting cofactors involved in this process will contribute to a better understanding of the action of IN during HIV-1 replication and aid in development of efficient and comprehensive anti-HIV strategies.

## Competing interests

The authors declare that they have no competing interests.

## Authors' contributions

YFZ, ZJA and KDJ constructed different IN mutants, and performed experiments and contributed to the writing of the manuscript. XJY designed, coordinated the study, and contributed to the writing of the manuscript. All authors read and approved the final manuscript.
